# Exercise modifies lipid and glucose metabolism alterations induced by sleep deprivation in mice

**DOI:** 10.5935/1984-0063.20220061

**Published:** 2022

**Authors:** Bruna Rafaele Diogenes da Silva, Paulo Iury Gomes Nunes, Flavia Almeida Santos, Pedro Felipe Carvalhedo de Bruin, Veralice Meireles Sales de Bruin

**Affiliations:** 1 Federal University of Ceará, Graduate Program in Medical Sciences, Department of Clinical Medicine - Fortaleza - Ceará - Brazil; 2 Federal University of Ceará, Graduate Program in Pharmacology, Department of Physiology and Pharmacology - Fortaleza - Ceará - Brazil

**Keywords:** Sleep Deprivation, Exercise, Glycogen, Lipids, Cholesterol, Liver

## Abstract

Insufficient sleep compromises lipid/glucose homeostasis. In opposition, exercise increases energy expenditure and has positive effects on glucose and fatty acid metabolism. Presently, it is hypothesized that exercise ameliorates metabolic dysfunction associated with sleep deprivation (SD). The effects of exercise (EX), SD and EX before SD. (EX+SD) on lipid and glucose metabolism were evaluated. Swiss mice were assigned to 4 groups (N=12, each) control, exercise (EX, 8 weeks, 1-hour of treadmill/9cm/s, 5x/week, from noon to 1:00 p.m.), SD (SD-72h, multiple platforms method), and exercise before SD (EX+SD). Exercise increased blood glucose, lactate and triglycerides (*p*<0.05). Both, SD and EX+SD reduced blood triglycerides (*p*<0.05). EX increased VLDL and reduced LDL; conversely, SD and EX+SD reduced VLDL and increased LDL. Hepatic triglycerides were markedly reduced by SD (*p*<0.05) and this was prevented by previous exercise (EX+SD). In summary, exercise improved essential cholesterol fractions and exercise before SD increased hepatic cholesterol and prevented hepatic triglycerides depletion.

## INTRODUCTION

An adequate sleep period and a balanced circadian rhythm influence the body’s hormonal and metabolic control and, therefore, are fundamental to human physiology. Sleep deprivation (SD) is frequent in modern society, having been associated with prevalent diseases, such as, hypertension, diabetes, and obesity^1^. Sleep deprivation is also habitual in working conditions associated with rotating shifts and extended working hours. Medical professionals, military operations and athletes involved in high-performance competitions are often exposed to extreme SD^2^. All this evidence shows that sleep restriction is common and has deleterious effects on human health. Given that exercise increases metabolism and has general beneficial impacts on human health, it is important to elucidate the effects of exercise, SD and exercise in association with SD.

Sleep restriction has been associated to insulin resistance, weight gain and increased risk of diabetes. Furthermore, insulin resistance seems to be key to alterations of glucose and lipid metabolism. For instance, hepatic steatosis, the most common hepatic disorder, is connected to SD and hepatic insulin resistance^3^. Shift work and night-work increase fasting blood glucose, glycated hemoglobin, triglycerides, low-density lipoprotein cholesterol (LDL-C), and lower high-density lipoprotein cholesterol (HDL-C) levels. These findings confirm the devastating effects of SD and circadian alterations on glucose and lipid metabolism.

Considerable evidence shows that exercise lowers blood glucose and reduces blood cholesterol, LDL-C, very low-density lipoprotein cholesterol (VLDL-C) and triglycerides while improving HDL-C^4^. It has been alleged that exercise combined with weight loss is more effective to improve metabolic measures. A discussion over the efficacy of aerobic exercise versus resistance training also exists^5^.

It has been previously reported that SD has a negative effect on exercise performance^6^. However, studies evaluating the impact of exercise on SD, particularly on metabolism, are scarce. Previously, a synergistic interaction effect between insufficient sleep and insufficient physical activity has been demonstrated^7^. Also, it has been shown that exercise reduces anxiety and cognitive impairment induced by SD^8^. High-intensity interval exercise training attenuated the detrimental impaired glucose tolerance associated with sleep loss^9^. Both, exercise and SD, probably have differential effects on specific organs and are influenced by preexisting or basic conditions and, in fact, the relationship between exercise with SD needs more clarification.

Studies using animal models give the opportunity to evaluate the effects of exercise and SD, and to examine the effects of exercise before SD on specific organs and tissues. The objective of this study was to evaluate lipid and glucose metabolism in plasma, liver and muscle, in mice submitted to chronic aerobic exercise before SD.

## MATERIAL AND METHODS

The experimental protocol was performed in Swiss mice (48 adult male, 25 to 30g) housed 6 per cage under standard conditions (light-dark cycle: 12h/12h, temperature: 23±1°C, 60% humidity) and free access to food and water throughout the entire study. Animals were distributed into four groups (N=12, each), as described: group (SD) was subjected to SD (72h -multiple platforms method), group (EX) performed up to one-hour of treadmill exercise (9cm/s, 5x/week, for 8 weeks, regularly from noon to 1p.m, and group (EX+SD) included mice that exercised (1h treadmill exercise for 8 weeks) before (72h - multiple platforms method).

The protocol used in the present study was in accordance with the ethical principles of animal experimentation established by the National Council for the Control of Animal Experimentation (CONCEA), and was previously approved by the Animal Use Ethics Committee of the Federal University of Ceará, No. 31/17.

### Sleep deprivation

Sleep deprivation was obtained using the multiple platform method adapted for mice^10^. Groups of 6 mice were placed on platforms in tanks (32cm×42cm×18cm) for 72 hours. Each tank contained 14 platforms (3cm in diameter) surrounded by water up to 1cm beneath the surface of the platforms. In this model, animals can move inside the tank by jumping from one platform to another and restriction of movement or social isolation are not imposed. During the experiments, food and water were available through a grid placed on top of the water tank. In this experimental model, when muscle atonia occurs during REM sleep, the animal contacts the water surrounding the platform and immediate awakening occurs.

### Exercise

For the exercise intervention, a rodent treadmill (Insight^®^ Model - Research and Education Equipment, Brazil) was used. Mice were subjected to adaptive treadmill exercise (progressive increase from 10 to 60 minutes for 2 weeks, speed 5-6cm/s, slope 0) followed by formal exercise training for six weeks, regularly from noon to 1PM. Exercise training was conducted for 60 minutes a day, 5 days a week, with a slope of 0. The speed was 5-6cm/s in weeks 1-2 and in weeks 3-8 the speed was 9cm/s. With the aim of avoiding the influence of environmental factors, the mice in the other groups were exposed to the same experimental environment during exercise training.

### Sample collection and analysis

Mice were euthanized by decapitation and, immediately after experimental procedure, a fresh blood sample of blood was obtained and placed on a test strip for glucose testing (Abbott’s FreeStyle Optium Neo^®^) (results in mg/dL, range 10 to 600mg/dL). Serum, liver and gastrocnemius muscle were collected at once and frozen at -80°C until analysis.

Glycogen determination was performed as described by Hassid and Abrahams (1957)^11^, adapting the methodology created by Sjögren et al. (1938)^12^. The tissue was mixed with 30% KOH, heated to 100°C for 30 min, and 95% ethanol was added. The pellet was mixed with distilled water and then added 0.2% anthrone (in 95% H_2_SO_4_), absorbance was measured at 490nm, and the data were plotted on a standard glucose curve. Liver lipid extraction was performed using the methodology described by Enternman et al. (1957)^13^, where the homogenization of 100mg of liver tissue in ethyl alcohol and ether solution (3:1) was performed. After heating at 65°C for 1h, the homogenate was centrifuged at 2000rpm for 10min. The supernatant was then separated and concentrated by evaporation, cooled to room temperature and diluted with 3mL isopropyl alcohol for subsequent use of total cholesterol and triglyceride quantitation assays according to their respective biochemical kits (Labtest^®^, Minas Gerais, Brazil). Lactate, triglyceride, total cholesterol and fractions (high density lipoprotein - HDL; low density lipoprotein - LDL; and very low density lipoprotein - VLDL) were analyzed in serum as described in Labtest^®^ commercial kits (Minas Gerais, Brazil), and the levels were expressed in mg/dL.

### Data analysis

Data are presented as mean ± standard error means (SEM). All data were tested for normal distribution and equal variance. Analysis of variance (ANOVA) followed by Newman-Keuls post-hoc test was employed to compare results between exercise, SD, exercise before SD and control. Comparisons between groups were made by using a two-tailed two-way analysis of variance (ANOVA two-way). Statistical significance was established at *p*<0.05.

## RESULTS

Results are expressed in detail in Table 1. Comparison between control, exercise, SD and exercise before SD shows that cholesterol blood levels were not different between groups. Exercise, as compared to control, increased blood glucose and lactate. It also increased triglycerides, HDL and VLDL, and decreased LDL. In addition, exercise increased hepatic and muscle glycogen.

**Table 1:** Summary of statistical data from blood, serum and tissue biochemical assessments.

	Control (mean, SD)	Exercise (mean, SD)	Sleep deprivation (mean, SD)	Exercise + Sleep deprivation (mean, SD)	ANOVA F/*p*-value
**Glucose (mg/dL)**	114.27 ± 6.28	148.40 ± 8.39 a,^*^	96.00 ± 5.68 a,b,^*^	99.45 ± 9.34 a,b,^*^	9.8/0.000
**Lactate (mg/dL)**	71.69 ± 4.57	98.50 ± 4.56 a,^**^	64.63 ± 2.79 b,^*^	77.80 ± 5.26 b,^*^	10.2/0.000
**Triglycerides (mg/dL)**	90.40 ± 5.66	149.50 ± 18.47 a,^**^	50.56 ± 3.79 a,b,^*^	54.03 ± 2.58 a,b,^*^	27.8/0.000
**Cholesterol (mg/dL)**	89.00 ± 3.28	93.78 ± 3.19	94.83 ± 4.96	96.33 ± 3.48	0.67/0.57
**HDL (mg/dL)**	49.71 ± 2.48	53.06 ± 1.55	52.35 ± 1.88	61.57 ± 3.39 a,b,c	4.4/0.009
**LDL (mg/dL)**	18.33 ± 1.35	9.85 ± 0.78 a,^**^	30.99 ± 2.36 a,b,^**^	21.88 ± 1.15 b,c	29.2/0.000
**VLDL (mg/dL)**	21.09 ± 2.43	30.89 ± 4.04 a,^**^	10.11 ± 0.76 a,b,^**^	13.28 ± 1.74 a,b,^*^	14.3/0.000
**Hepatic glycogen (mg/100mg)**	3.00 ± 0.26	4.77 ± 0.35 a,^*^	2.57 ± 0.04 b,^**^	2.73 ± 0.04 b,^**^	22.2/0.000
**Muscle glycogen (mg/100mg)**	0.59 ± 0.01	0.65 ± 0.01 a,^*^	0.62 ± 0.01 b,^*^	0.62 ± 0.01 b,^*^	8.2/0.000
**Hepatic cholesterol (mg/100mg)**	2.37 ± 0.13	2.87 ± 0.17	2.77 ± 0.15	3.84 ± 0.48 a,b,c	4.7/0.007
**Hepatic triglycerides (mg/100mg)**	15.78 ± 1.8	13.28 ± 0.50	8.32 ± 0.95 a,b,^**^	20.72 ± 2.67 a,b,c	9.8/0.000

Notes: HDL = High density lipoprotein; LDL = Low density lipoprotein; VLDL = Very low density lipoprotein.

a *p*≤0.05, statistical comparison with the control group;

b *p*≤0.05, statistical comparison with the EX group;

c *p*≤0.05, statistical comparison with the SD group. Student-Newman-Keuls post Hoc test;

* *p*≤0.05;

** *p*≤0.01.

Sleep deprivation, as compared to control and exercise, reduced blood glucose; and as compared to exercise, decreased lactate. Additionally, SD reduced triglycerides and VLDL, and, increased LDL. Sleep deprivation reduced hepatic and muscle glycogen as compared to exercise, and reduced hepatic triglycerides as compared to control and exercise groups.

Exercise before SD, as compared to control and exercise groups, reduced blood glucose, triglycerides and VLDL. Exercise before SD reduced lactate, hepatic and muscle glycogen as compared to exercise. Exercise before SD also increased HDL as compared to SD and hepatic cholesterol and triglycerides as compared to all other groups.

## DISCUSSION

The main findings of this study are the modifying, and, sometimes, opposing effects of SD and exercise on lipid and glucose metabolism. The effects of exercise increasing blood glucose, lactate levels, triglycerides, HDL, and VLDL are largely confirmed by the literature^4^,^14^. Sleep deprivation was associated with a consumption of glucose, lactate, triglycerides and VLDL, and an unfavorable increase in LDL. In short, exercise prevented the consumption of hepatic triglycerides associated with SD and increased hepatic cholesterol levels.

Previously, hepatic lipid homeostasis, particularly cholesterol, has been formerly demonstrated to be essential for survival in situations of stress, e.g., hibernation and thermal injury. The liver, with its metabolic, inflammatory, immunological and acute phase functions, creates a closed system in which the efficient use of lipoproteins is essential for the organism’s survival and recovery. Dysregulation of this system can lead to several life-threatening^15^. Other works have corroborated the essential role of hepatic cholesterol as a powerhouse or a last source of energy in situations of stress^16^. The present findings suggest that exercise before SD had a protective role in maintaining the essential storage of glycogen, triglycerides, and cholesterol.

With further regard to the effects of exercise on metabolism and in agreement with previous literature, exercise increased serum glucose, lactate, and triglycerides^14^,^17^. Our results also confirm that both, SD and EX before SD, markedly reduced serum glucose and triglycerides reaffirming the alterations in metabolism induced by SD^18^. An association of SD with higher LDL and lower HDL and, by contrast, the association of exercise with lower LDL and higher HDL substantiate the occasional opposing actions of exercise and SD^19^. These results concerning the beneficial effects of exercise on metabolism are of interest considering that SD is associated with unfavorable insulin resistance that contributes to unwanted overweight/obesity^3^.

It is accepted that exercise exerts both acute and chronic effects on plasma lipid and lipoprotein concentrations. For instance, it has been reported that only prolonged exercise augments plasma triglyceride clearance^19^. Classically, aerobic exercise combined with low energy diet reduces weight, waist circumference, cholesterol, and triglycerides levels^20^. Interestingly, in this experiment, serum cholesterol was largely unaltered in all groups. As regards glucose metabolism, it has been shown that in unchallenged and basic conditions, exercise initially increases glucose and insulin sensitivity and after some time it derives energy from free fat acids and reduces glucose and insulin activity^21^. Beyond dispute, cumulative evidence indicates that, chronically, exercise is a potential tool for a reduction of glucose that is much wanted in diabetic patients^22^.

In conclusion, this work consolidates the beneficial effects of exercise improving essential cholesterol fractions and reducing LDL, while SD mostly led to an opposite direction. Importantly, exercise prevented the consumption of hepatic triglycerides associated with SD and increased hepatic cholesterol levels that are essential as storage depot for metabolism.
